# Chronic exposure to warm temperature causes low sperm abundance and quality in *Drosophila melanogaster*

**DOI:** 10.1038/s41598-023-39360-7

**Published:** 2023-07-30

**Authors:** Ana Caroline P. Gandara, Daniela Drummond-Barbosa

**Affiliations:** 1 Department of Genetics, University of Wisconsin–Madison, Madison, WI 53706 USA; 2 Morgridge Institute for Research, Madison, WI 53706 USA

**Keywords:** Cell biology, Developmental biology, Genetics, Physiology, Stem cells

## Abstract

Temperature influences male fertility across organisms; however, how suboptimal temperatures affect adult spermatogenesis remains understudied. In a recent study on *Drosophila melanogaster* oogenesis, we observed a drastic reduction in the fertility of adult males exposed to warm temperature (29 °C). Here, we show that males become infertile at 29 °C because of low sperm abundance and quality. The low sperm abundance at 29 °C does not stem from reduced germline stem cell or spermatid numbers, as those numbers remain comparable between 29 °C and control 25 °C. Notably, males at cold 18 °C and 29 °C had similarly increased frequencies of spermatid elongation and individualization defects which, considering the high sperm abundance and male fertility measured at 18 °C, indicate that spermatogenesis has a high tolerance for elongation and individualization defects. Interestingly, the abundance of sperm at 29 °C decreases abruptly and with no evidence of apoptosis as they transition into the seminal vesicle near the end of spermatogenesis, pointing to sperm elimination through an unknown mechanism. Finally, sperm from males at 29 °C fertilize eggs less efficiently and do not support embryos past the first stage of embryogenesis, indicating that poor sperm quality is an additional cause of male infertility at 29 °C.

## Introduction

Reproduction is highly responsive to physiological and external signals^1–3^ in consequence of billions of years of evolution—initially of single cells and later of multicellular organisms that had to successfully produce progeny in the context of ever-changing environments^4,5^. Among the unintended effects of rapidly rising temperatures due to human activity^6^, however, are negative impacts on the reproduction of many organisms^7–15^. Effects on insects are particularly concerning given their limited capacity to thermoregulate^11–15^ and their public health, economical, and ecological relevance^16^.

Elevated temperatures have well documented negative effects on male fertility in many *Drosophila* species and other insects^17–34^. For example, *Drosophila melanogaster* larvae that develop at 28–31 °C give rise to adult males with lower fertility^17,19,21–24,26,30–34^, as is also the case for other drosophilids^20,25,27,29,35^. Short-term exposure of adult *Drosophila* males to temperatures above 37 °C also reduces progeny numbers^36,37^. Interestingly, a recent study using 43 *Drosophila* species showed that temperatures leading to adult male sterility predict their global distribution better than lethal temperatures, underscoring the key role that spermatogenesis can have on ecological processes^13^. Notably, suboptimal temperatures (as opposed to extreme temperatures) used in laboratory experiments are thought to more closely approximate climate change conditions in the wild than those involving extreme temperatures^11,15^. Despite the large number of studies reporting effects of temperature on male fertility^15,22,24,26–29,31,33^, how adult exposure to chronic suboptimal temperatures affects spermatogenesis in *Drosophila* remains unclear.

**Figure 1:**
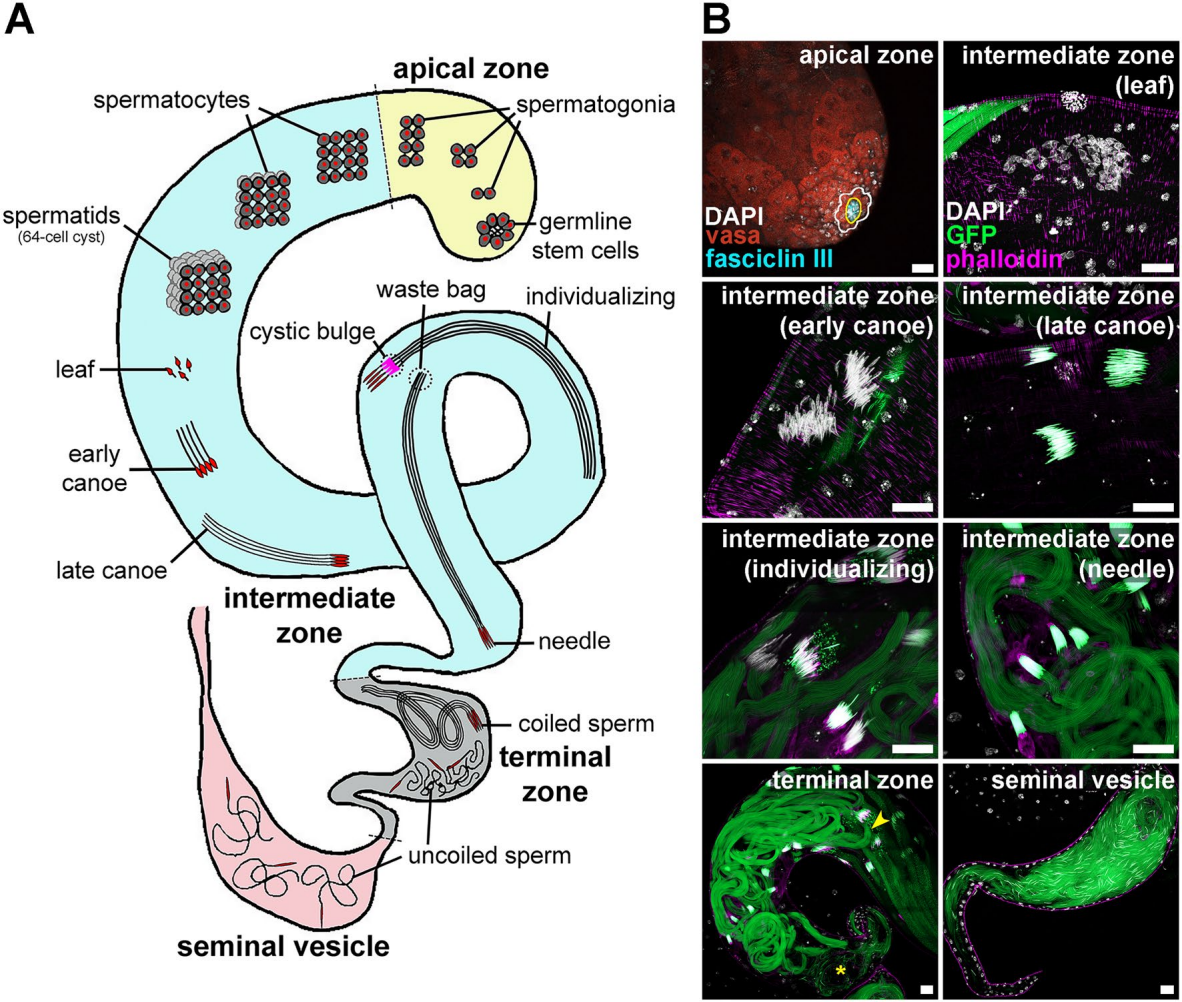
*Drosophila melanogaster* spermatogenesis. (**A**) Simplified testis diagram (not to scale) showing different stages of germ cell development, with germ cell nuclei shown in red. In the apical zone (yellow), germline stem cells (GSCs) divide asymmetrically to form gonialblasts (not shown) that further divide to form interconnected spermatogonia (ultimately in 16-cell cysts). In the intermediate zone (blue), cysts of 16 primary spermatocytes undergo meiosis I and II to form 64-cell cysts of haploid spermatids that undergo spermiogenesis. These syncytial spermatids first start elongating while their nuclei undergo chromatin compaction and morphological changes from round to leaf-, canoe- and, finally, needle-shaped. Spermatids undergo individualization with the help of an actin-based complex (magenta), forming a cystic bulge; at the end of individualization, organelle and cytoplasmic remains are discarded in a waste bag at the end of the spermatid tails, and each sperm is encased in its own plasma membrane. In the terminal zone (gray), bundles of mature sperm initially coil and are then released as uncoiled sperm prior to transfer to the seminal vesicle (light pink) for storage until fertilization. (**B**) Images illustrating different spermatogenesis stages. Vasa (red), germ cells; fasciclin III (cyan), hub cells; DAPI (white), nuclei; phalloidin (magenta), actin; GFP (green), Protamin B (labels germ cell nuclei starting in late canoe stage) and Don Juan (labels sperm tails). In the apical zone, hub cells and GSCs are outlined in yellow and white, respectively. In the terminal zone, an arrowhead indicates the beginning of the area with coiled sperm, while an asterisk indicates uncoiled sperm. Scale bars, 20 μm.

*Drosophila melanogaster* is a powerful system to investigate fundamental aspects of spermatogenesis of broad relevance to many organisms, including other insects^3,38–40^. The apical zone of the testis houses mitotically dividing germline stem cells (GSCs) and spermatogonia^41,42^ (Fig. 1). Five to nine GSCs surrounding the hub (a somatic niche) divide asymmetrically to self-renew and generate a gonialblast, which in turn divides four times with incomplete cytokinesis to form a germline cyst (surrounded by two somatic cyst cells) containing 16 spermatogonia that will develop into primary spermatocytes^38,43^. Meiosis and spermiogenesis (i.e., sperm differentiation) occur in the intermediate zone^41^ (Fig. 1A). Primary spermatocytes undergo two meiotic divisions to generate 64-cell cysts (i.e., 64 syncytial haploid spermatids). Their nuclei undergo morphological changes from round to leaf-, canoe-, and ultimately needle-shaped as they become elongating spermatids (Fig. 1); by late canoe, histones are replaced by protamines^39,40^. For example, Protamine B (encoded by *Mst35B*) is essential for proper nuclear morphology^44,45^; *Mst35B* is transcribed in round spermatocytes but remains translationally repressed until the late canoe stage^44^ (Fig. 1B). After nuclear condensation, needle-shaped spermatids become individualized through a caspase-dependent process involving actin-based individualization cones that strip away unnecessary organelles and cytoplasm, forming a cystic bulge as they move from head to tail to discard remains in a waste bag at the end of the cyst^46,47^. Following individualization, the resulting sperm will coil at the terminal zone (also known as coiling region)^41,48^ (Fig. 1). Sperm then uncoil and translocate to the seminal vesicle, where they are stored until mating (Fig. 1). We recently showed that *Drosophila melanogaster* males chronically exposed as adults to the suboptimal temperature of 29 °C (in contrast to the optimal temperature of 25 °C) become sterile^49^. However, precisely what spermatogenesis processes were negatively affected by warm temperature remained unknown.

Here, we analyze the effects of chronic exposure of adult *Drosophila* males to cold or warm temperatures on spermatogenesis and show that male sterility at 29 °C is caused by reduced sperm production and low sperm quality. Chronic exposure of males to 18 °C (cold) improved GSC maintenance, while males at 25 °C and 29 °C had similar GSC numbers. Surprisingly, despite the high cyst numbers and fertility maintained at 18 °C, males at both 18 °C and 29 °C had increased elongation and individualization defects, indicating a high tolerance of spermatogenesis for these types of defects. At 29 °C, sperm numbers drastically decreased between the terminal zone and seminal vesicle with no sign of apoptosis, suggesting that sperm are eliminated through an as-yet-unknown mechanism at the end of spermatogenesis. Finally, we show that the fewer sperm produced after chronic exposure of males to 29 °C have low quality, as indicated by their lower fertilization efficiency and failure to support embryogenesis past stage 1. Our findings provide a solid foundation for future studies addressing the cellular and molecular mechanisms underpinning the deleterious effects of warm temperatures on the production of healthy sperm.

## Results

### *Drosophila* male fertility drops within five to 10 days at 29 °C

**Figure 2:**
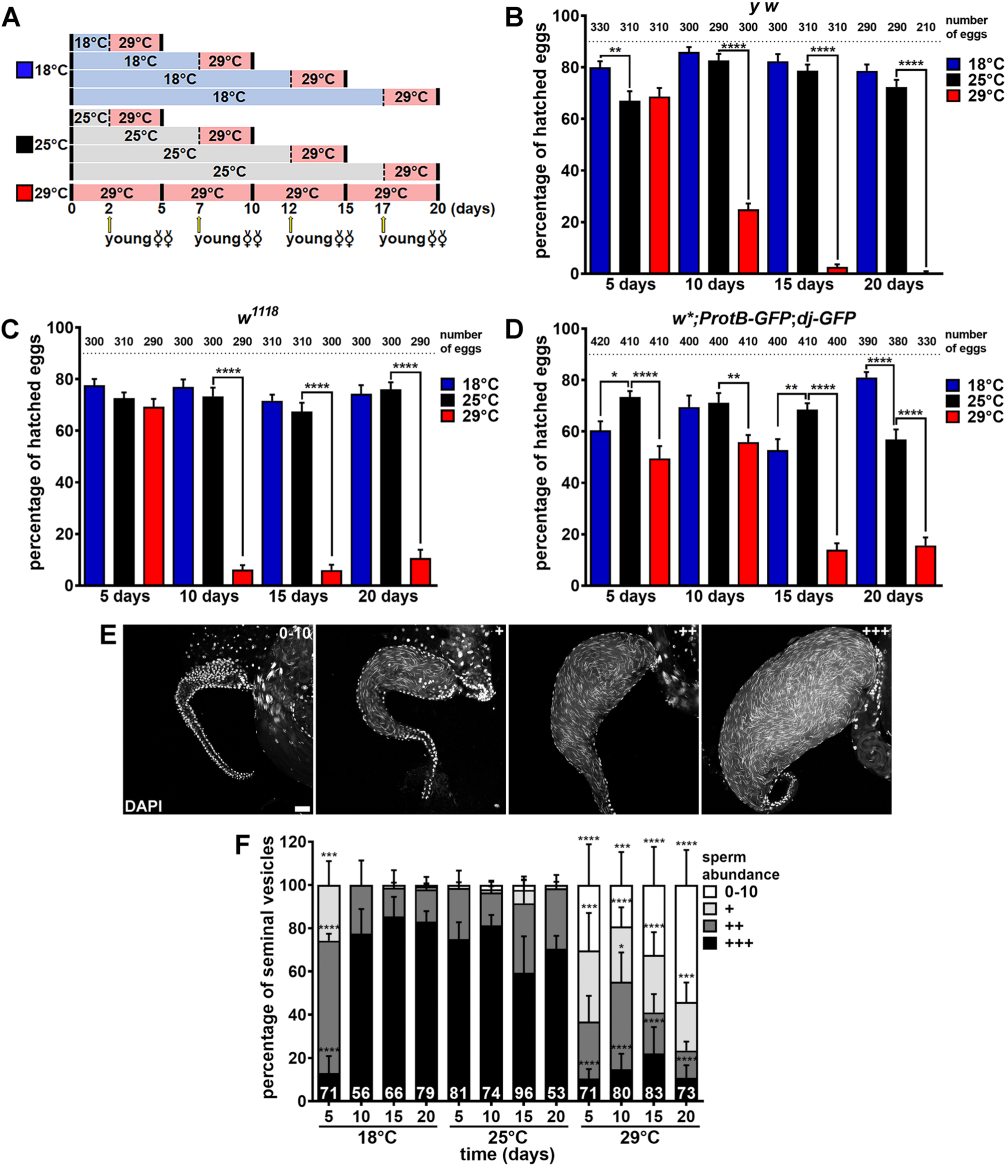
Males exposed to warm temperature become infertile and have seminal vesicles with fewer sperm. (**A**) Experimental design for hatching rate experiments in (**B**–**D**). Couples were incubated at 18 °C, 25 °C or 29 °C for two, seven, 12 or 17 days, after which the original females were replaced with two-day-old virgin females (arrows) and incubated (with original males) for three additional days at 29 °C (see Methods for details). (**B**–**D**) Percentage of hatched eggs laid by females within the last 24 h of incubation with *y w* (**B**), *w*^*1118*^ (**C**), or w*; ProtB-GFP; dj-GFP (**D**) males. The number of eggs analyzed are shown above bars. Data shown as mean ± S.E.M. from three or four independent experiments. *P < 0.05, **P < 0.01, ****P < 0.0001, one-way ANOVA using 25 °C as control (Supplementary Information 2). (**E**) Seminal vesicles showing different sperm abundances to illustrate 0–10 sperm, +, ++, and +++ categories quantified in (**F**). DAPI (white), nuclei. Scale bar, 20 μm. (**F**) Frequencies of seminal vesicles in different sperm abundance categories from *y w* males maintained at 18 °C, 25 °C, or 29 °C for five, 10, 15, or 20 days. Numbers of seminal vesicles analyzed are shown inside bars. Data shown as mean ± S.E.M. from four independent experiments. *P < 0.05, ***P < 0.001, ****P < 0.0001, Chi-square test using 25 °C as control (Supplementary Information 2).

We previously showed that *Drosophila y w* “wildtype” males maintained at 29 °C for 20 days are nearly 100% sterile^49^. We therefore asked how rapidly *y w* males lose their fertility at 29 °C and whether similar loss of fertility at 29 °C is observed in other “wildtype” strains, namely *w*^*1118*^ and *ProtB-GFP; dj-GFP* (which produce GFP-labeled sperm; see Methods). We incubated couples at 18 °C, 25 °C, or 29 °C for two, seven, 12, or 17 days, and then substituted two-day-old virgin females (previously at 25 °C) for the original females before an additional three-day incubation at 29 °C, for a total of five-, 10-, 15- or 20-day incubation at 29 °C for males (Fig. 2A). To assess male fertility, we measured the hatching rates of eggs laid by these young females within the last 24 h at 29 °C as previously described^49^. *y w* and *w*^*1118*^ males showed a drastic drop in fertility after 10 days of incubation at 29 °C compared to males at the 25 °C control temperature (Fig. 2B, C). The fertility of *ProtB*-GFP; *dj*-GFP males was slightly reduced at five and 10 days, and a more severe drop was observed at later time points at 29 °C relative to 25 °C (Fig. 2D). Male fertility remained similar or higher at 18 °C compared to 25 °C at most time points, except that *ProtB*-GFP; *dj*-GFP males had slightly lower fertility at 18 °C compared to 25 °C at 5 and 15 days (Fig. 2B–D), which is possibly related to strain differences^26^. Nonetheless, *ProtB*-GFP; *dj*-GFP males were consistently more fertile at 18 °C than at 29 °C at all time points (Fig. 2D). These results show that while chronic exposure to cold temperature is mostly not detrimental to male reproduction, chronic exposure of males to warm temperature causes a significant reduction in their fertility within five to 10 days.

### Reduced sperm numbers contribute to the loss of male fertility at 29 °C

The drastic reduction in male fertility at 29 °C led us to wonder if fewer sperm were being produced. We therefore quantified sperm abundance in the seminal vesicles of *y w* males maintained at 18 °C, 25 °C, or 29 °C for five, 10, 15, or 20 days (Fig. 2E, F). In males at 25 °C, sperm abundance remained high at all time points, while males at 18 °C had lower abundance at five days but were comparable to 25 °C controls from 10 days on (Fig. 2F). By contrast, when males were incubated at 29 °C, sperm numbers were significantly reduced within five days and, by 20 days, over half of the seminal vesicles analyzed had only zero to ten sperm (Fig. 2F). These results indicate that reduced sperm production partially explains the reduced fertility of males at 29 °C.

### The numbers of germline stem cells and 64-cell cysts remain similar in males maintained at 25 °C and 29 °C, but are elevated in males at 18 °C

**Figure 3:**
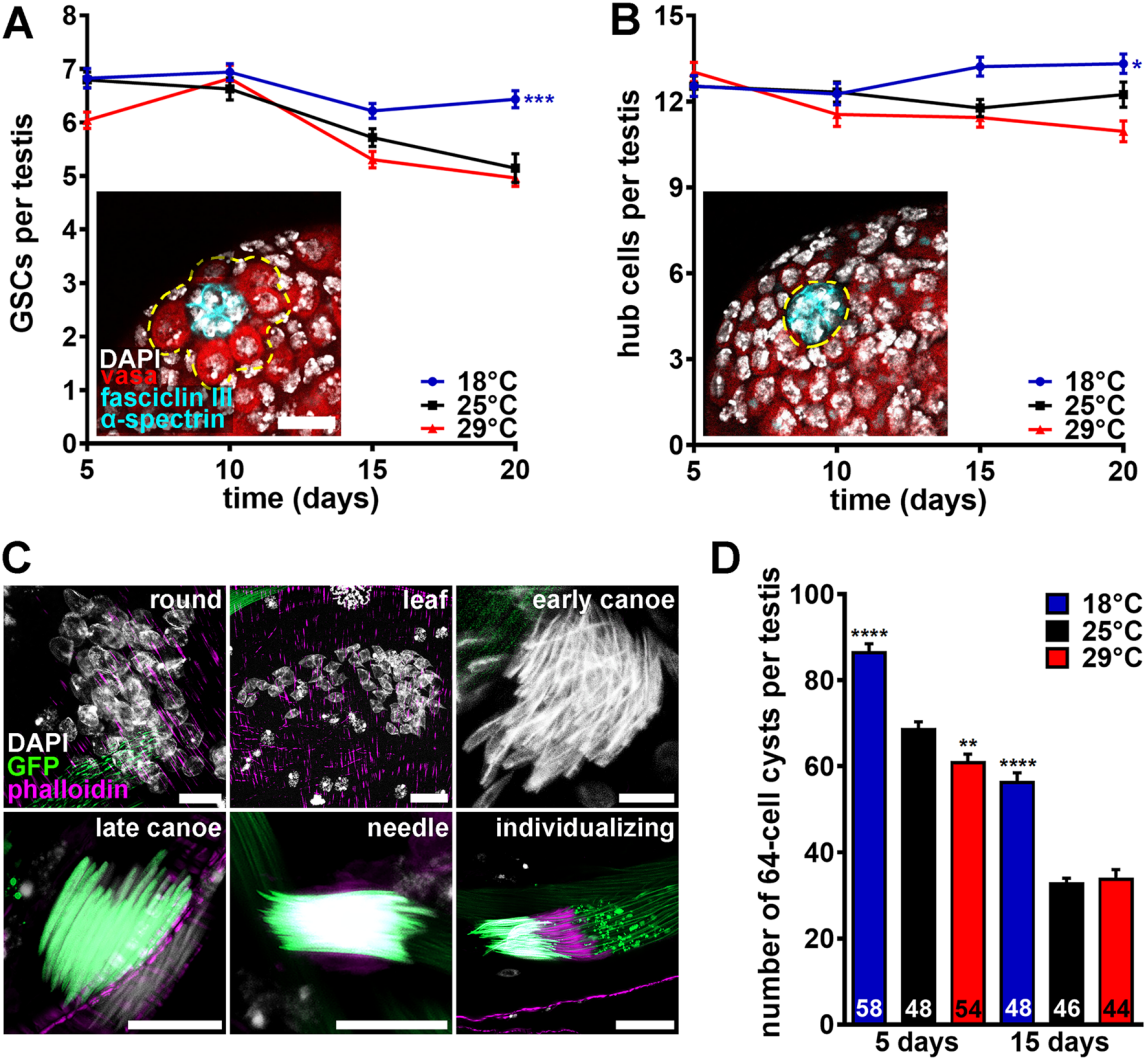
Males at cold temperature maintain higher numbers of germline stem cells and 64-cell cysts. (**A**, **B**) Average numbers of GSCs (**A**) or hub cells (**B**) per testis in *y w* males maintained at 18 °C, 25 °C, or 29 °C for five, 10, 15, or 20 days. Images show testis apexes from males at 25 °C for 5 days. Fasciclin III (cyan), α-spectrin (cyan), fusomes; hub cells; vasa (red), germ cells; DAPI (white), nuclei. GSC and hub cells are outlined in (**A**) and (**B**), respectively. Scale bar, 15 μm. Numbers of testes analyzed are included in Supplementary Data S1. Data shown as mean ± S.E.M. from four independent experiments. *P < 0.05, ***P < 0.001, two-way ANOVA with interaction using 25 °C as control (Supplementary Information 2). (**C**) Examples of 64-cell cysts in different stages of spermiogenesis from samples used for quantification in (**D**). DAPI (white), nuclei; phalloidin (magenta), actin; GFP (green), Protamin B (labels germ cell nuclei starting in late canoe stage) and Don Juan (labels sperm tails). Scale bar, 10 μm. (**D**) Average number of 64-cell cysts per testis in w*; ProtB-*GFP; dj*-*GFP* males maintained at 18 °C, 25 °C, or 29 °C for five or 15 days. Numbers of testes analyzed are shown inside bars. Data shown as mean ± S.E.M. from four independent experiments. **P < 0.01, ****P < 0.0001, one-way ANOVA using 25 °C as control (Supplementary Information 2).

We next asked whether changes in GSC numbers in the apical zone or developing spermatids in the intermediate zone (Fig. 1) might account for the reduced sperm production at 29 °C. We counted the number of GSCs in males maintained at different temperatures for five, 10, 15 or 20 days. GSC numbers declined at similar rates in males at 25 °C or 29 °C but remained higher over time at 18 °C (Fig. 3A). We did not observe a proportional difference in the number of hub cells (Fig. 3B), suggesting that differences in GSC numbers are largely independent of changes in niche size. These results parallel our findings for female GSCs at different temperatures^49^. We quantified the total number of 64-cell cysts at any stage of spermatid differentiation (Fig. 3C) along the intermediate zone and found they were similar at 25 °C and 29 °C (Fig. 3D). Interestingly, 64-cell cyst numbers remained higher at 18 °C over time, perhaps in part as a consequence of higher GSC numbers (Fig. 3D; also see Supplementary Fig. S1 in Supplementary Information 1 for representative images of *ProtB-GFP; dj-GFP* testes at different temperatures). Altogether, these results demonstrate that the lower sperm production at 29 °C is not caused by changes in the numbers of GSC or developing spermatids.

### Spermiogenesis defects do not fully explain the decreased sperm abundance at 29 °C

**Figure 4:**
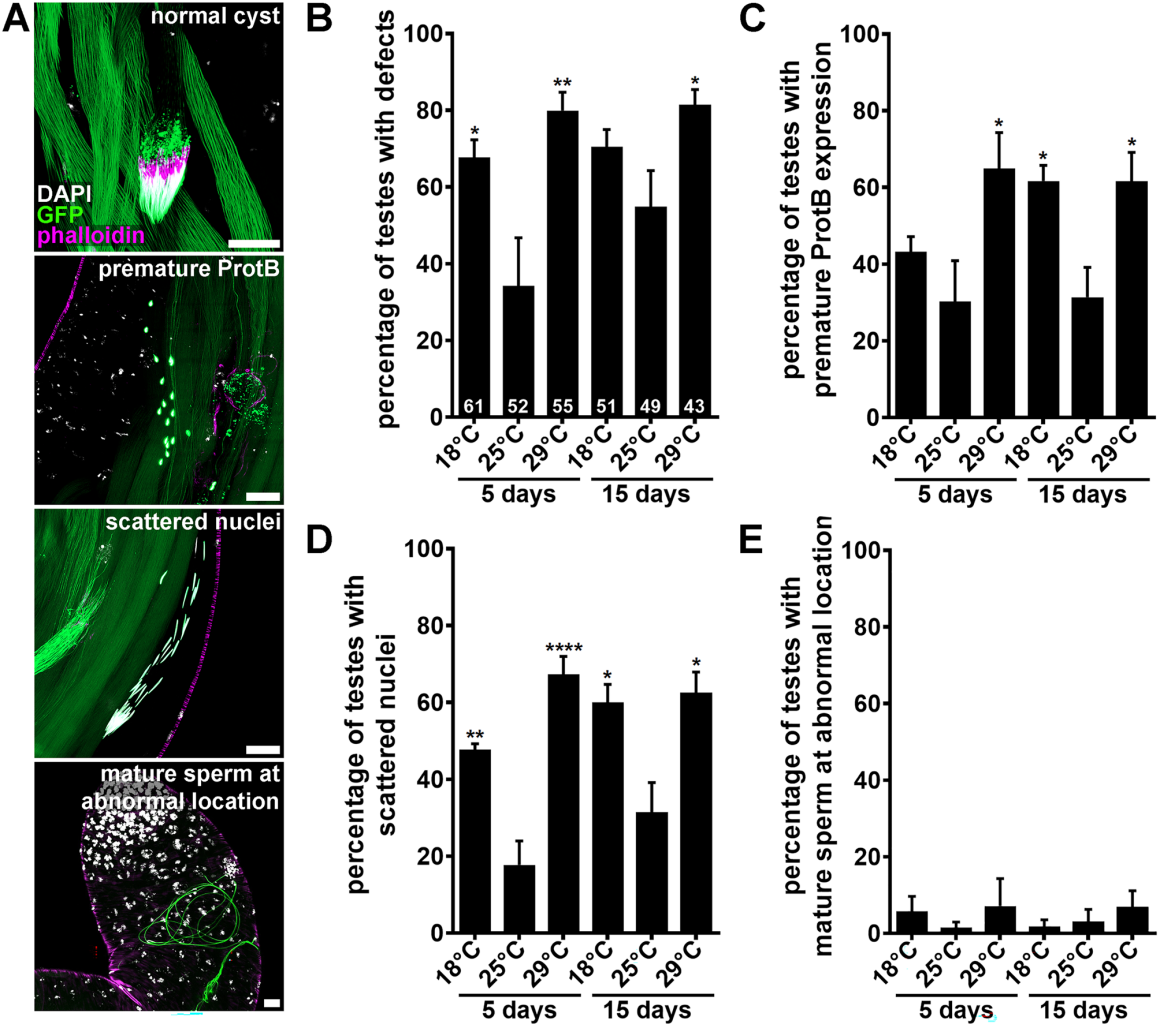
Chronic exposure to either 18 °C or 29 °C causes higher incidence of spermiogenesis defects. (**A**) Images showing examples of a normal cyst of spermatids (top panel) or clusters of spermatids showing premature Protamine B expression or scattered nuclei (middle panels), or abnormally localized mature sperm (bottom panel). DAPI (white), nuclei; phalloidin (magenta), actin; GFP (green), Protamin B (labels germ cell nuclei starting in late canoe stage) and Don Juan (labels sperm tails). Scale bars, 20 μm. (**B**–**E**) Percentage of testis from w*; ProtB-GFP; dj-GFP males maintained at 18 °C, 25 °C, or 29 °C for five or 15 days showing any defects (**B**), premature Protamine B expression in round, leaf, or early canoe stages (**C**), scattered nuclei (**D**), or abnormally localized sperm (**D**). Numbers of testes analyzed are shown inside bars in (**A**). Data shown as mean ± S.E.M. from four independent experiments. *P < 0.05, **P < 0.01, ****P < 0.0001, one-way ANOVA using 25 °C as control (Supplementary Information 2).

Considering that 64-cell cyst numbers were not affected at 29 °C, we wondered if defects in spermiogenesis might account for the reduced number of sperm in seminal vesicles. We noticed atypical spermatids along the testes at all temperatures, including round, leaf, and early canoe stages prematurely expressing Protamine B (i.e. “premature *ProtB*”), cysts with “scattered nuclei”, and mature sperm abnormally located more anteriorly (Fig. 4A). Intriguingly, the overall percentage of testes containing defective spermatids was significantly elevated in males maintained at 18 °C or 29 °C relative to 25 °C (Fig. 4B), and the predominant defects were “premature *ProtB*” and “scattered nuclei” (Fig. 4C–E). In view of the normal sperm abundance at 18 °C (Fig. 2E, F), the similar increase in the number of testes with abnormal spermatids at 18 °C and 29 °C suggests that spermatid defects do not fully explain the decrease in sperm abundance in seminal vesicles of males chronically exposed to 29 °C, although we cannot rule out more subtle differences between 18 °C and 29 °C defects. These results further show that adult males can tolerate high levels of spermatid differentiation defects while maintaining normal sperm production and fertility. Accordingly, naturally occurring defects during spermatogenesis are commonly observed in human seminal fluid^50^. Although another study reported decreased *Drosophila* adult male fertility at 18 °C, those flies had also been raised at 18 °C^26^, and development at 17 °C has been shown to reduce adult fertility^34^.

### Sperm are eliminated by an apoptosis-independent mechanism between the terminal zone and seminal vesicle in males at 29 °C

**Figure 5:**
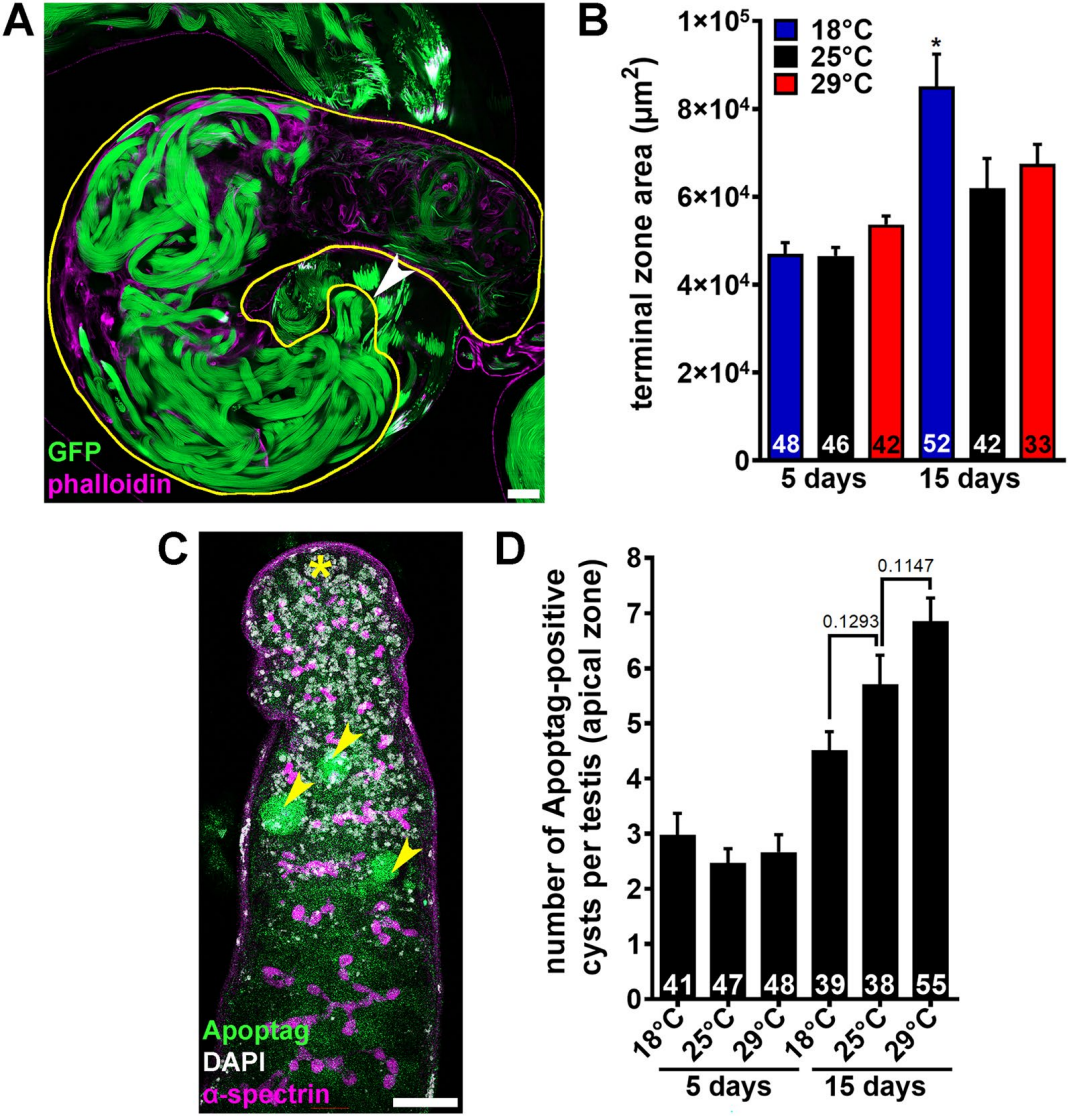
Males exposed to warm temperatures have normal-sized terminal zones and show normal numbers of apoptotic cells in the apical zone. (**A**) Terminal zone in the testis from a w*; ProtB-GFP; dj-GFP male. Phalloidin (magenta), actin; GFP (green), Protamin B (sperm nuclei) and Don Juan (sperm tails). The terminal zone is outlined in yellow as done for quantification (see “Methods”). The arrowhead indicates the beginning of the terminal zone region containing coiled cysts. Scale bar, 20 µm. (**B**) Average terminal zone area in w*; *ProtB-GFP*; *dj-GFP* males maintained at 18 °C, 25 °C, or 29 °C for five or 15 days. Numbers of terminal zones analyzed are shown inside the bars. Data shown as mean ± S.E.M. from three independent experiments. *P < 0.05, one-way ANOVA using 25 °C as control (Supplementary Information 2). (**C**) Testis apex showing cysts of dying spermatogonia (arrowheads). DAPI (white), nuclei; Apoptag (green), dying germ cells; α-spectrin (magenta), fusomes (i.e., specialized organelles present in early germ cells). Asterisk indicates hub. Scale bar, 20 μm. (**D**) Number of Apoptag-positive cysts per testis from *y w* males incubated for five or 15 days at 18 °C, 25 °C or 29 °C. Numbers of testes analyzed are shown inside bars. Data shown as mean ± S.E.M. from four independent experiments. No statistically significant differences, one-way ANOVA using 25 °C as control (Supplementary Information 2).

To assess whether large numbers of sperm might be eliminated during transfer from the intermediate zone to the terminal zone at 29 °C, we measured the size of the terminal zone in males exposed to different temperatures for five or 15 days (Figs. 1, 5A). The area of the terminal zone was comparable at all temperatures at five days (Fig. 5B). At 15 days, there was no significant difference in the size of the terminal zone in males at 25 °C or 29 °C (Fig. 5B). The terminal zone was larger in males at 18 °C (Fig. 5B), consistent with their larger number of spermatid cysts (Fig. 3C, D). The normal size of the terminal zone in 29 °C males (Fig. 5A, B) and the drastic reduction in sperm abundance in their seminal vesicles (Fig. 2E, F) support the conclusion that sperm are eliminated during their transition between the terminal zone and the seminal vesicle.

To test if sperm are eliminated by apoptosis at 29 °C, we stained testes using a TUNEL assay (Apoptag), which labels DNA breaks for detection of apoptotic cells, including sperm^51^. We observed statistically similar numbers of Apoptag-positive cysts in the apical zone (see Fig. 1) of all testes regardless of the temperature, with higher numbers at 15 days compared to five days (Fig. 5C, D). Indeed, apoptotic germ cells are frequently observed in the mitotic zone as they are spontaneously eliminated before entering meiosis^52,53^. In stark contrast, we did not observe Apoptag-positive cysts at any of the later stages (i.e., intermediate zone, terminal zone, and seminal vesicle; see Fig. 1) in any of the testes analyzed from males at any temperature (n = 41–55 testes; see Fig. 5D). These results suggest that the sperm elimination between the terminal zone and seminal vesicle at 29 °C occurs through an apoptosis-independent mechanism, thereby explaining the reduction in sperm levels by the end of spermatogenesis.

### Sperm motility and transfer to females are not specifically affected in males at 29 °C

**Figure 6:**
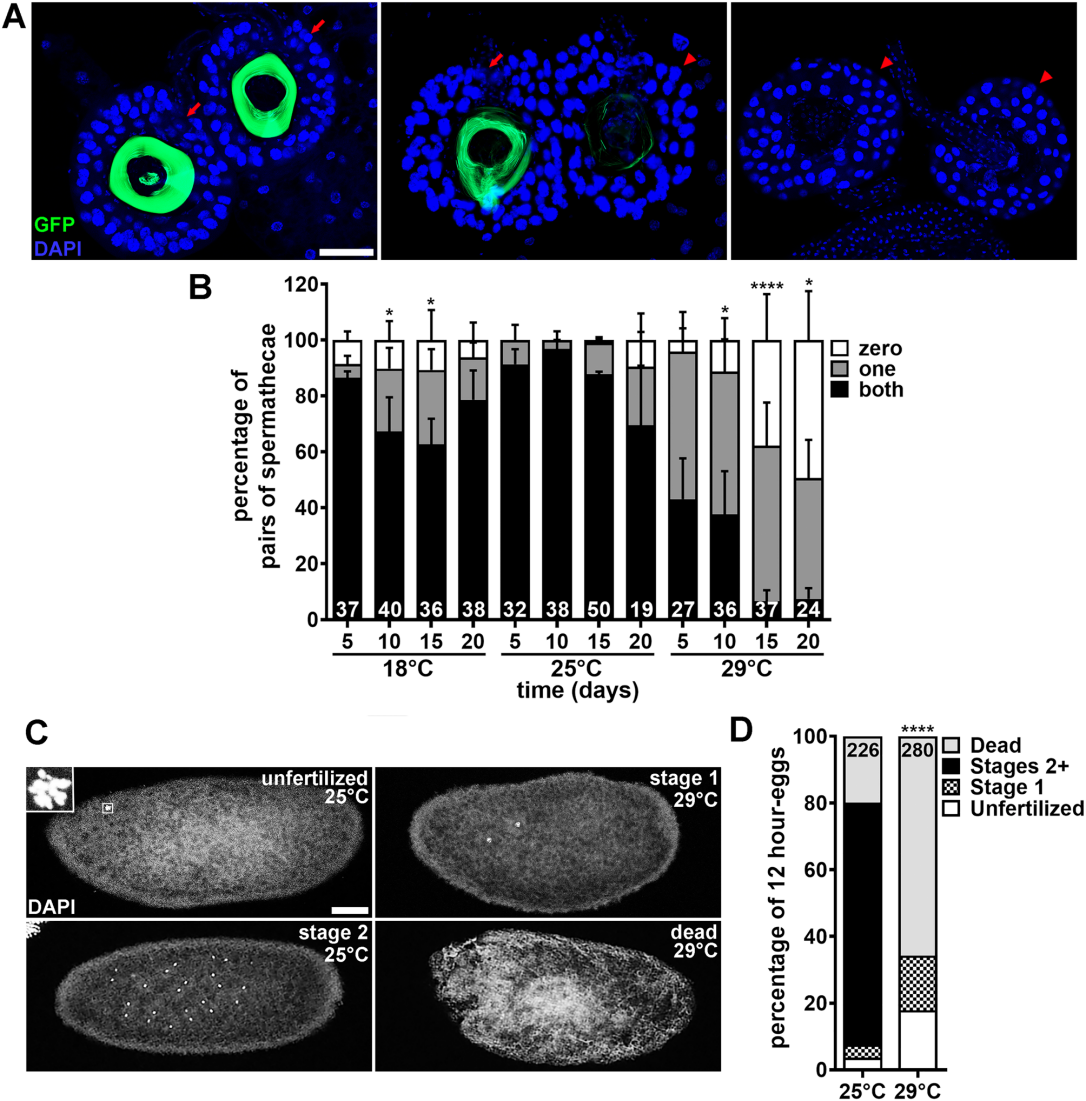
Sperm from males exposed to warm temperature have reduced fertilization efficiency and fail to support early embryo development. (**A**) Examples of spermatheca pairs in which both (left panel), only one (middle panel), or neither (right panel) contain sperm transferred from w*; ProtB-GFP; dj-GFP males used for quantification in (**B**). GFP (green), Protamin B (sperm nuclei) and Don Juan (sperm tails); DAPI (blue), spermatheca nuclei. Arrows indicate spermathecae containing sperm. Arrowheads indicate empty spermathecae. Scale bar, 50 µm. (**B**) Percentage of spermatheca pairs in which neither, one, or both spermathecae contain sperm. Numbers of spermatheca pairs analyzed are shown inside bars. Data shown as mean ± S.E.M from four independent experiments. *P < 0.05, ****P < 0.0001, Chi-square test using 25 °C as control (Supplementary Information 2). (**C**) Examples of 12-h collection eggs used for quantification in (**D**). For egg collection, *y w* couples were incubated for 17 days at 25 °C or 29 °C, after which the original females were replaced with two-day-old virgin females and incubated (with original males) for three additional days at 29 °C. Images show unfertilized egg, containing rosette (outlined by square and magnified in inset); stage 1 embryo, containing two nuclei; stage 2 embryo, containing multiple centrally-located nuclei; dead, amorphous material with no recognizable nuclei. Scale bar, 50 µm. (**D**) Percentage of eggs from 12-h collection representing unfertilized eggs, stage 1 embryos, stage 2 or later embryos, or dead material. Numbers of embryos analyzed are shown inside bars. Data represent one large experiment. ****P < 0.0001 (for each of the four categories), Chi-square test using 25 °C as control (Supplementary Information 2 ).

The sharp drop in male fertility at 29 °C (Fig. 2A–D) is only partially explained by the reduction in sperm abundance in their seminal vesicles (Fig. 2E, F). For example, *y w* males maintained at 29 °C for 20 days are completely sterile (Fig. 2B) even though a significant percentage of seminal vesicles still contain sperm in these males (Fig. 2F). Elevated temperatures can lead to decreased sperm motility in both mammals^54,55^ and insects^27,35,56^. We therefore tested if males at 29 °C could transfer sperm from their seminal vesicles to spermathecae (one of the sperm storage organs in females^57^) by taking advantage of the green sperm from *ProtB-GFP; dj-GFP* males^58^. *ProtB-GFP; dj-GFP* males were maintained at 18 °C, 25 °C or 29 °C for two-, seven-, 12- or 17-days. Two-day-old *y w* virgin females were substituted for previous females for three additional days of incubation at 29 °C (see Fig. 2A), after which we quantified the presence of sperm in their spermathecae. Nearly all females paired with males previously incubated at either 18 °C or 25 °C (regardless of the time point) had sperm in one or both spermatheca (Fig. 6A, B). By contrast, females paired with 29 °C males showed a progressive reduction in sperm-containing spermathecae over time (Fig. 6B), consistent with our seminal vesicle sperm abundance results (Fig. 2E, F). Notably, 100% of spermathecae with sperm showed normal sperm motility regardless of temperature (Supplementary Table S1 and Supplementary Movies S1–S3 in Supplementary Information 1), further suggesting that sperm transfer from males to females is not specifically affected by temperature. These findings rule out problems with sperm motility or transfer as potential causes of the severe loss of male fertility at 29 °C.

### Sperm quality is severely reduced in males chronically exposed to 29 °C

We next hypothesized that the drop in male fertility beyond what can be attributed to reduced sperm abundance might be the result of poor sperm quality (e.g., inefficient fertilization and/or reduced ability to support embryo development). To test this hypothesis, we incubated *y w* males (with females) at 25 °C or 29 °C for 17 days, at which point two-day-old virgin *y w* females (previously at 25 °C) were substituted for original females prior to incubation for three additional days at 29 °C (see Fig. 2A). We then analyzed eggs collected within the last 12 h at 29 °C for fertilization status and embryonic development using DAPI to label nuclei (Fig. 6C). Unfertilized eggs were identified based on the “rosette” formed by condensed female pronucleus and polar bodies near their surface^59,60^. Developing embryos were staged as described^61,62^; for example, stage 1 begins after fertilization and ends once the first two zygotic divisions have been completed, while divisions 3–8 occur during stage 2^61,62^. Eggs containing amorphous material without recognizable nuclei were classified as “dead”. Approximately 3.5% of the eggs laid by females incubated with 25 °C males had “rosettes” while that frequency rose to 18% for females paired with 29 °C males (Fig. 6C, D; Supplementary Table S2 in Supplementary Information 1), suggesting that 29 °C sperm fertilize eggs less efficiently. Remarkably, all the remaining eggs from females incubated with 29 °C males contained either stage 1 embryos (16%) or were “dead” (66%), while most control eggs contained embryos at various stages of development (77%) (Fig. 6C, D; Supplementary Table S2 in Supplementary Information 1). These results show that sperm from males maintained at 29 °C for 20 days are not able to support embryogenesis past stage 1.

## Discussion

**Figure 7:**
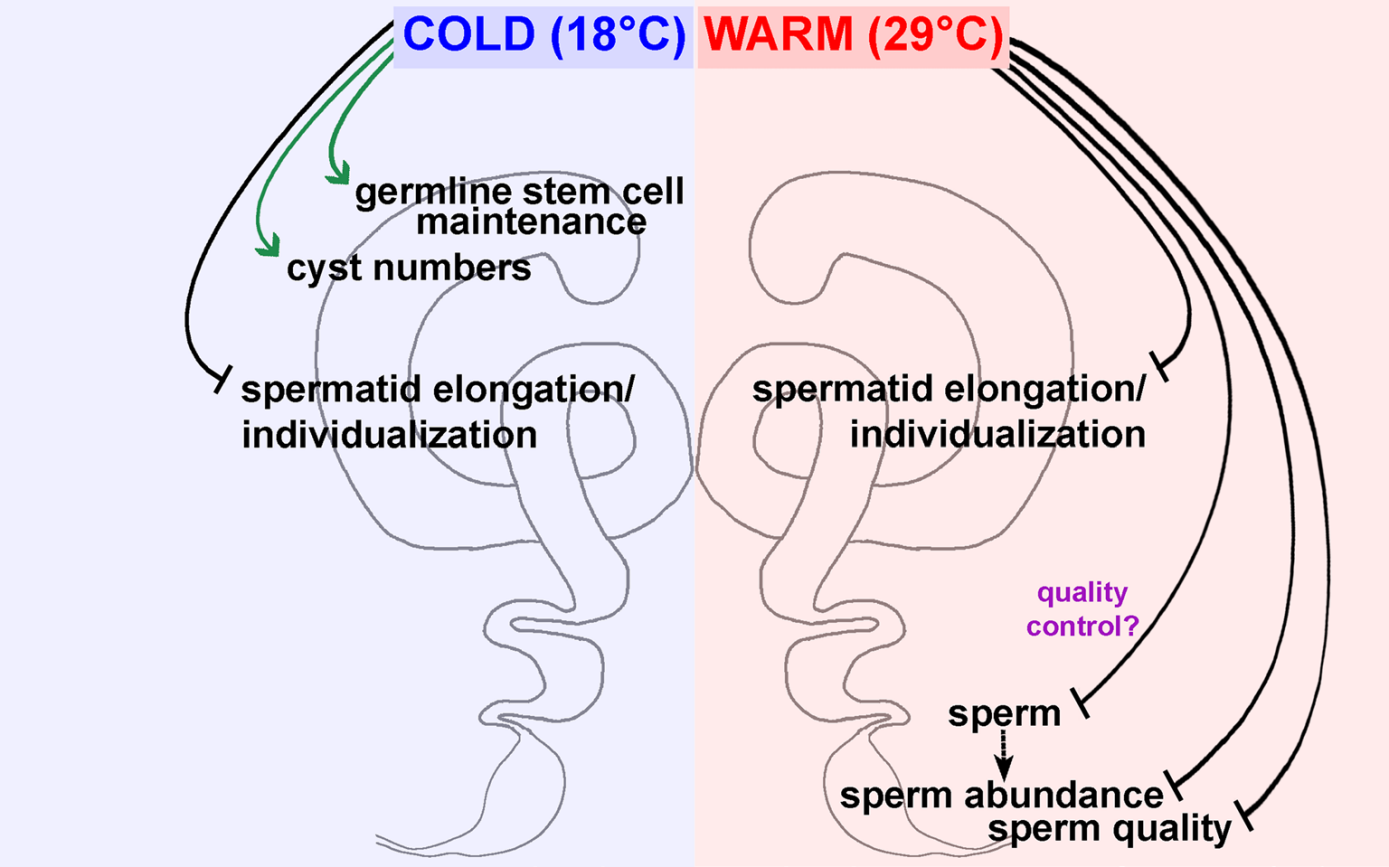
Model for how chronic exposure to warm, but not cold, temperature causes adult male infertility in *Drosophila melanogaster*. Chronic exposure of adult males to either cold (18 °C) or warm (29 °C) temperature increases the incidence of spermatid elongation and individualization defects despite normal sperm abundance and quality at 18 °C. In males at 18 °C, the numbers of GSCs and 64-cell cysts are higher than at 25 °C. By contrast, males at 29 °C become infertile as a combination of reduced sperm numbers and quality. Sperm abundance is reduced at 29 °C through an unknown, apoptosis-independent process of sperm elimination at the transition between the terminal zone and the seminal vesicle. This sperm elimination might be random or represent a quality-control process.

Anthropomorphic climate change has caused natural disasters, weather extremes, and environmental degradation^6^, posing a major risk to the reproduction of many organisms, including humans^8,10^. Insects face a greater challenge due to their limited capacity for thermoregulation^11–15^. In fact, sublethal temperatures have begun altering insect distribution through effects on their fertility, and these effects are strong predictors of insect extinction^11,13,14^. Despite the vast literature on how temperature affects larval gametogenesis^17,19,21–24,26,29–34^, the cellular effects of chronic exposure of adult insects to suboptimal temperatures have remained largely understudied. Complementing our recent findings on how temperature affects adult oogenesis^49^, this study shows that chronic exposure of males to cold (18 °C) does not affect their fertility, while warm temperature (29 °C) leads to a significant reduction in sperm abundance and quality (Fig. 7). We find that low sperm abundance results from elimination of sperm through an unknown mechanism at the end of spermatogenesis (prior to transfer to seminal vesicles) and that sperm are inefficient at fertilizing eggs and cannot support embryo development. Our findings provide a foundation for future studies in *Drosophila melanogaster* to investigate the cellular and molecular mechanisms underlying sperm elimination and the inability of sperm to support the early embryo when males are chronically exposed to warm temperature.

These findings also contribute towards a detailed understanding of the effects of suboptimal temperatures on insect spermatogenesis more generally. For example, warm temperatures also reduce adult sperm abundance and fertility of another insect model, *Tribolium castaneum*^28^. In various species of *Drosophila* and one species of wasp, exposure to warm temperature during larval stages results in later adults with fewer sperm in their seminal vesicles and reduced fertility^29,31,35,63^. Our new data further add to recent discussions highlighting the urgency of investigating how sublethal temperatures impact insect reproduction^6,11,13–15^.

Beyond insects, a deeper understanding of how *Drosophila melanogaster* spermatogenesis responds to warm temperatures is potentially relevant to mammals. Despite being warm-blooded, mammals are susceptible to high environmental temperatures, fever, heat stroke, and thermoregulatory disorders^64,65^. Testes are particularly sensitive, as testicular temperatures are normally maintained at 2–4 °C lower than core temperature to ensure optimal spermatogenesis^50^. In fact, heat stress resulting from climate change is a major factor for decreased fertility and production of livestock, and heat disruption of testicular thermoregulation drastically reduces bovine fertility and semen quality^66^. Multiple studies in mice, rats, and cattle have shown that paternal heat stress reduces sperm abundance and pregnancy rates^50,55,67–69^. In humans, high temperatures have multiple effects on spermatogenesis, including morphological changes, decreased sperm count, and increased DNA damage^54,55,64,65,68–72^. Future studies comparing specific findings in *Drosophila* and mammals would help us understand the extent of commonality in how different systems handle the stress of elevated temperatures.

An important question raised by our studies is how sperm are eliminated between the terminal zone and seminal vesicle in response to warm temperature in *Drosophila*. Under normal conditions, quality control mechanisms have been proposed to eliminate defective spermatids during elongation and individualization^46^ or coiling^48^. In the case of warm temperatures, however, it remains unknown whether sperm is randomly eliminated or a third quality control mechanism is operating at the transition between the terminal zone and seminal vesicle.

The low sperm quality at 29 °C could potentially result from any number of defects in early events surrounding fertilization^73^. Following sperm entry and activation, the needle-shaped, protamine-rich, and highly condensed sperm nucleus needs to be converted into a male pronucleus capable of DNA replication. This process involves the removal of protamines followed by the assembly of paternal genome-wide nucleosomes from maternal histones and nucleosome assembly factors. Histone acetylation and methylation marks are also differentially distributed along paternal and maternal chromosomes, and assembly of the male pronuclear envelope occurs by the onset of pronuclear migration^73^. Sperm centrioles combined with pericentriolar material from the egg generate zygotic centrosomes that are crucial for the formation of the sperm aster, which is in turn required for migration of the female pronucleus towards the male pronucleus. Once pronuclei juxtapose each other, the first zygotic division begins, initiating the transition to rapid embryonic cycles^73^. Our findings that sperm from 29 °C males are not able to support embryogenesis past stage 1 suggest that any of these early processes might be impaired when sperm develop at warm temperatures. Future studies carefully examining early events around fertilization and known molecular players will be critical to pinpoint the reason for the poor quality of 29 °C sperm. A deeper understanding of how suboptimal temperatures affect insect reproduction may pave the way for the development and adoption of appropriate interventions and improvement of public health, agricultural, and environmental policies.

## Materials and methods

### *Drosophila* strains and culture conditions

Fly stocks were maintained at 21–23 °C on standard medium containing 4.64% w/v cornmeal, 5.8% v/v molasses, 1.74% w/v yeast, and 0.93% w/v agar. *y*^*1*^* ac*^*1*^* w*^*1118*^ (referred here as *y w*), *w*^*1118*^, and w*; *ProtamineB-eGFP/CyO; dj-GFP/TM3, Sb*^*1*^^58^ were obtained from the Bloomington Drosophila Stock Center (bdsc.indiana.edu). For most experiments, zero-to-one-day-old adults were incubated in standard medium supplemented with dry yeast at 18 °C (± 0.5), 25 °C (± 0.5) or 29 °C (± 0.5) for five, 10, 15, or 20 days at ≥ 80% humidity in 12 h:12 h Light–Dark cycles (Darwin Chambers incubators), except where noted. Food was changed and temperature and humidity were monitored daily.

### Egg collections for quantification of hatching rates and analysis of embryo development

Male fertility was measured based on egg hatching rates. To measure hatching rates, we incubated 10 zero- to one-day-old males of different genotypes with 10 *y w* females for two, seven, 12, or 17 days at 18 °C, 25 °C, or 29 °C in vials. Two-day-old virgin *y w* females (previously at 25 °C) were then substituted for the older females and incubated with original males for three additional days at 29 °C for a total of five-, 10-, 15-, or 20-day incubation, respectively (see Fig. 2A) in perforated bottles closed with molasses/agar plates covered by a thin layer of wet yeast paste, as previously described^49^. For hatching rates, eggs were collected over the last 24 h of incubation at 29 °C. Around 100 eggs were placed in groups of 10 on molasses plates containing a dab of yeast paste at the center and incubated in a humid chamber (covered Pyrex dish lined with wet paper towels) for 24 h at 25 °C. The unhatched eggs were counted and subtracted from the total to calculate the number of hatched eggs. Data from three (for *y w* and *w*^*1118*^ males) or four (for *ProtB*-*GFP*; *dj*-*GFP* males) independent experiments were statistically analyzed using one-way ANOVA (GraphPad Prism) with 25 °C as control (Supplementary Information 2 ).

For analysis of embryo development, *y w* adults were prepared as described above, except that eggs were collected over the last 12 h of incubation at 29 °C (only for the 20-day time point) and immediately processed as previously described^74^, with minor modifications. Briefly, eggs were rinsed with distilled water and dechorionated with 50% bleach for two min. After washing with distilled water, they were shaken vigorously for two min in 1:1 heptane:methanol. After three washes in methanol, samples were fixed overnight at 4 °C in methanol and gradually rehydrated in PBS (10 mM NaH_2_PO_4_/NaHPO_4_ and 175 mM NaCl (pH 7.4). After five min of incubation in PBST (PBS plus 0.1% Triton X-100), embryos were mounted in Vectashield containing DAPI (Vector Labs) for microscopy (see below). Data from one large experiment was subjected to a Chi-square test (Microsoft Excel) (Supplementary Information 2).

### Tissue immunostaining and fluorescence microscopy

Testes were dissected in Grace’s Medium, fixed for 30 min at room temperature in fixing solution [5.3% formaldehyde (Ted Pella) in Grace’s medium], rinsed and washed three times for 15 min each in PBST. After one hour incubation in blocking solution [5% normal goat serum (MP Biochemicals) plus 5% bovine serum albumin (Sigma) in PBST], testes were incubated overnight at 4 °C in the following primary antibodies diluted in blocking solution: mouse monoclonal anti-α-Spectrin (3A9) [Developmental Studies Hybridoma Bank (DSHB), 1:20]; mouse monoclonal anti-Fasciclin III (7G10) (DSHB, 1:50); and rat monoclonal anti-vasa (DSHB, 1:20). Testes were rinsed and washed as above and incubated for two hours at room temperature with Alexa Fluor 488- or 568-conjugated secondary antibodies (Molecular Probes, 1:400) in blocking solution. For spermiogenesis analysis, testes from *ProtB*-*GFP*; *dj*-*GFP* adult males were dissected, fixed, washed (as above) and incubated for 20 min in 5U/mL phalloidin 568 (Invitrogen) in PBS at room temperature and protected from light instead. Spermathecae from *y w* females incubated with *ProtB*-*GFP*; *dj*-*GFP* males were dissected and fixed as above. All samples were rinsed, washed two times for 15 min each in PBST, and mounted in Vectashield containing DAPI (Vector Labs). All images were collected on an LSM 900 confocal system using the integrated Airyscan 2 detector and corresponding Airyscan SR mode (ZEISS Microscopy) and processed with the standard Airyscan Processing utilities of the ZEN Blue 3.5 software (ZEISS Microscopy) to achieve super-resolution readouts.

### Apoptag TUNEL assay

The ApopTag Fluorescein Direct In Situ Apoptosis Detection Kit (S7160, Millipore Sigma) was used to identify dying cells as previously described^49,53^. Data from four independent experiments were subjected to one-way ANOVA (GraphPad Prism), using 25 °C as control (Supplementary Information 2).

### Quantification of germline stem cells, hub cells, 64-cell cysts, and elongation and individualization defects

Hub cells were identified based on Fasciclin III-positive staining, and GSCs were identified as vasa-positive cells immediately surrounding the hub^42^. Data from four independent experiments were subjected to two-way ANOVA with interaction (GraphPad Prism), using 25 °C as control (Supplementary Information 2). The total number of 64-cell cysts in all developing stages (from round through needle stage spermatids; see Fig. 3C) were counted along the testes. The same testes were used for quantification of elongation and individualization defects and mislocalized mature sperm described in results. Data from four independent experiments were subjected to one-way ANOVA (GraphPad Prism), using 25 °C as control (Supplementary Information 2).

### Measurement of terminal zone size

We identified the terminal zone based on the presence of coiled cysts at its beginning and border with seminal vesicle at the end (see Fig. 5A and Supplementary Fig. S1 in Supplementary Information 1). We outlined the terminal zone in the middle optical section (for the largest cross-section) using Freehand selection by ImageJ (Fiji) and measured its area using the Measure tool. Data from three independent experiments were subjected to one-way ANOVA (GraphPad Prism), using 25 °C as control (Supplementary Information 2).

### Analysis of sperm abundance in seminal vesicles and of sperm presence and motility in spermathecae

We quantified sperm abundance in seminal vesicles of testes stained with DAPI (see Fig. 2E). To classify each seminal vesicle into a specific category (0–10, +, ++, or +++), we carefully examined each DAPI-stained seminal vesicle throughout the Z-plane using the 20 × objective lens at the LSM 900 confocal microscope. In the "0–10" category, seminal vesicles contained 10 or fewer sperm (and both "sides" of the seminal vesicle epithelium were typically observed in the same focal plane); in the "+" category, seminal vesicles did not have quite enough sperm to fill the space between both "sides" of the seminal vesicle epithelium (and smaller portions of both "sides" of the seminal vesicle epithelium could be observed in the same focal plane); in the "++" category, there was enough sperm to completely fill the space between both "sides" of the seminal vesicle, but the vesicle was not yet fully distended; in the "+++" category, the seminal vesicle was so full of sperm that it bulged and had a more rounded appearance. For seminal vesicle quantification, data from four independent experiments were subjected to a Chi-square test (Microsoft Excel), using 25 °C as control (Supplementary Information 2).

We quantified the percentage of females in which both spermathecae had sperm, only one spermatheca had sperm, or neither spermatheca had sperm; any spermatheca with fewer than 10 sperm was considered as having no sperm. To assess sperm motility, freshly dissected spermathecae were transferred to a slide with Grace’s medium and immediately visualized using an AxioZoom.V16 (ZEISS Microscopy). Samples were analyzed using Plan Z 1 × objective, X-Cite Xylis Lamp, 38 HE eGFP reflector, and Exc. 450–490 nm/Em. 500–550 nm filters. Videos were recorded by Axio 712 mono through ZEN Blue 3.5 software (ZEISS Microscopy). For spermatheca quantification, data from four independent experiments were subjected to a Chi-square test (Microsoft Excel), using 25 °C as control (Supplementary Information 2).

## Supplementary Information

Supplementary Information 1.

Supplementary Information 2.

Supplementary Video 1.

Supplementary Video 2.

Supplementary Video 3.

## Data Availability

All the raw data from this study are available on request.
