# Supplementary material for: Chronic exposure to warm temperature causes low sperm abundance and quality in Drosophila melanogaster
Source: Sci Rep. 2023 Jul 30;13:12331. doi: 10.1038/s41598-023-39360-7 (PMC10387475; doi:10.1038/s41598-023-39360-7)
Supplement: Supplementary file 1 — Supplementary Information 1. [file 41598_2023_39360_MOESM1_ESM.pdf]

Supplemental material for

Chronic exposure to warm temperature causes low sperm abundance and quality in *Drosophila melanogaster*

Ana Caroline P. Gandara[1,2] and Daniela Drummond-Barbosa[1,2]*

[1]Department of Genetics, University of Wisconsin – Madison, Madison, WI, 53706, USA

[2]Morgridge Institute for Research, Madison, WI, 53706, USA

*Corresponding author: ddbarbosa@wisc.edu

SUPPLEMENTAL FIGURE

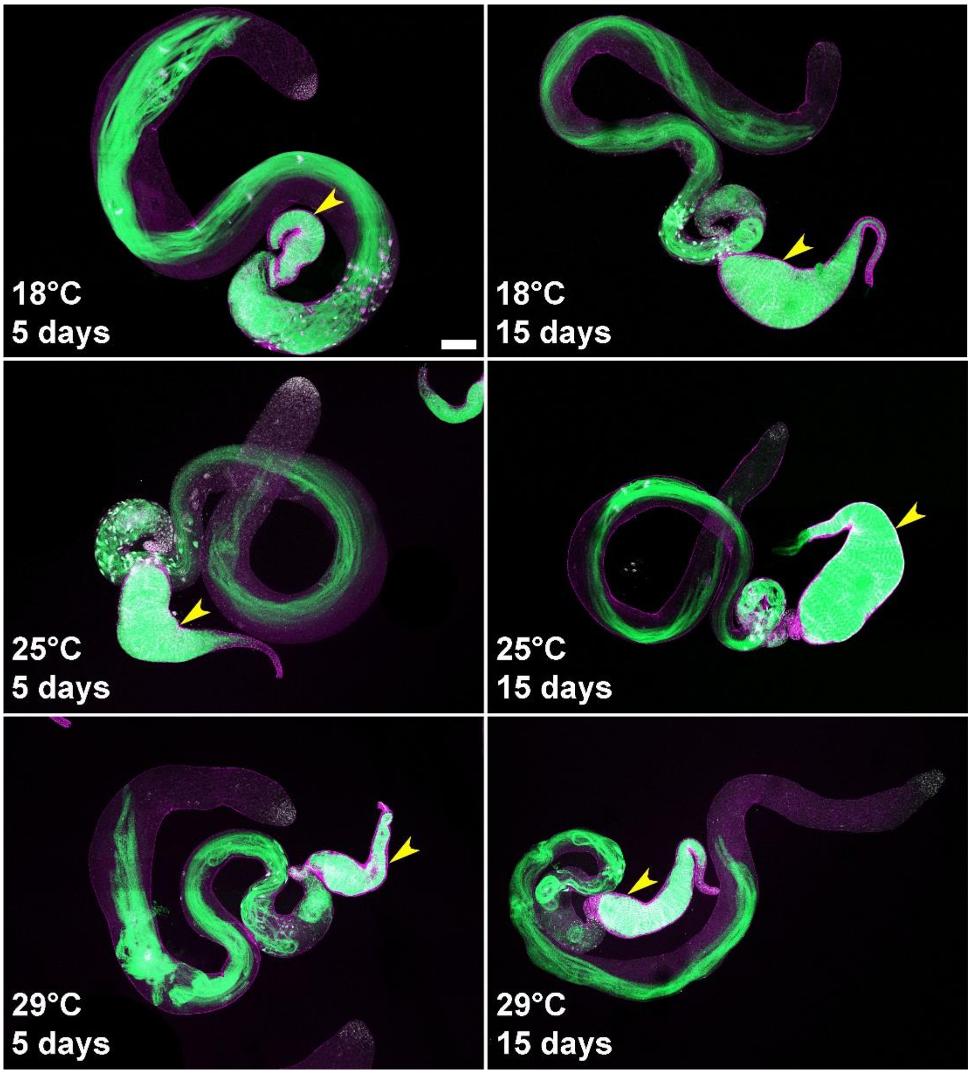

Figure S1. Whole testes from males maintained at different temperatures.

Representative whole testes images from *w*; ProtB-GFP; dj-GFP* males incubated at 18°C, 25°C, or 29°C for five or 15 days used for analysis shown in Fig. 3C,D.

Phalloidin (magenta), actin; GFP (green), Protamin B (labels germ cell nuclei starting in late canoe stage) and Don Juan (labels sperm tails); DAPI (white), nuclei.

SUPPLEMENTAL TABLES

Table S1. Numbers of spermatheca pairs analyzed for sperm motility

Temperature	5 days*	10 days*	15 days*	20 days*
18°C	10	16	14	22
25°C	7	17	25	12
29°C	9	17	13	18

***100% of sperm-containing spermathecae had motile sperm.

Table S2. Numbers of eggs in different categories from 12-hour collection testing effects of temperature on sperm quality

Temperature	Dead*	Stage 1*	Stage 2+*	Unfertilized*	Total
25°C	45<br>(20)[#]	9<br>(4)	164<br>(73)	08<br>(3.5)	226
29°C	184<br>(66)	46<br>(16)	0<br>(0)	50<br>(18)	280

*These categories are explained in the text and Fig. 6C, and these same data are graphed in Fig. 6D.

[#]Percentages are shown in parentheses.

SUPPLEMENTAL MOVIE LEGENDS

Movie S1. Representative spermatheca pair containing sperm from *w*; ProtB-GFP; dj-GFP* males maintained at 18°C for 20 days. Sperm show normal motility in both spermathecae (arrows) and in seminal receptacle (asterisk). GFP (green), Protamin B (sperm nuclei) and Don Juan (sperm tails).

Movie S2. Representative spermatheca pair containing sperm from *w*; ProtB-GFP; dj-GFP* males males maintained at 25°C for 20 days. Sperm show normal

motility in both spermathecae (arrows) and in seminal receptacle (asterisk). GFP (green), Protamin B (sperm nuclei) and Don Juan (sperm tails).

Movie S3. Representative spermatheca pair with sperm from *w*; ProtB-GFP; dj-GFP* males maintained at 29°C for 20 days. Sperm show normal motility in both spermathecae (arrows) and in seminal receptacle (asterisk). The spermatheca on the left has fewer than 10 spermatozoa. GFP (green), Protamin B (sperm nuclei) and Don Juan (sperm tails).
